# MicroRNA-497 Induces Apoptosis and Suppresses Proliferation via the Bcl-2/Bax-Caspase9-Caspase3 Pathway and Cyclin D2 Protein in HUVECs

**DOI:** 10.1371/journal.pone.0167052

**Published:** 2016-12-05

**Authors:** Ridong Wu, Shi Tang, Mian Wang, Xiangdong Xu, Chen Yao, Shenming Wang

**Affiliations:** 1 Department of Vascular Surgery, The First Affiliated Hospital of Sun Yat-sen University, Guangzhou, P. R. China; 2 Department of Breast Surgery, Dongguan Maternal & Children Health Hospital, Dongguan, P. R. China; Qatar University College of Health Sciences, QATAR

## Abstract

**Introduction:**

MicroRNAs play crucial roles in various types of diseases. However, to date, no information about the role of miR-497 in the development of atherosclerosis has been reported. This study investigated the possible role of miR-497 in vascular endothelial cell injury during the early stage of atherosclerosis.

**Materials and Methods:**

The expression level of miR-497 in human umbilical vein endothelial cells (HUVECs) exposed to ox-LDL was detected using qRT-PCR. To perform gain of function and loss of function analyses, miR-497 mimics were transfected into HUVECs, and miR-497 inhibitors were transfected into HUVECs stimulated with ox-LDL. Flow cytometry was used to analyze cell cycle progression and apoptosis. EdU and CCK-8 assays were employed to detect DNA synthesis and cell proliferation, respectively. After bioinformatics prediction, a dual Luciferase Reporter assay was used to analyze the direct target genes of miR-497. The mRNA and protein levels of the target genes were detected using qRT-PCR and western blot analyses, respectively. Caspase-9/3 activity was analyzed to determine the mechanism of endothelial dysfunction.

**Results:**

We showed that miR-497 was significantly upregulated in HUVECs stimulated with ox-LDL. Ectopic expression of miR-497 suppressed cell proliferation, induced apoptosis and increased the activity of caspase-9/3. After verification, Bcl2 and CCND2 were shown to be direct target genes of miR-497 in HUVECs. MiR-497 significantly suppressed cell proliferation by arresting the cell cycle through the CCND2 protein and induced apoptosis through the Bcl2/Bax-caspase9-caspase3 pathway.

**Conclusion:**

Overall, our study shows that miR-497 might play a role in the development of atherosclerosis by inducing apoptosis and suppressing the proliferation of vascular endothelial cells. Therefore, miR-497 could be a potential therapeutic target for the treatment of atherosclerosis.

## Introduction

Atherosclerosis is a disease of the cardiovascular system that is characterized by chronic inflammation of the arterial wall[1]. Endothelial cells (ECs) play an important role in maintaining the homeostasis of the vascular system[2]. Current evidence has suggested that the initial qualitative change in the development of atherosclerosis is the injury of the endothelial cells, which line the inner wall of the arteries[3]. Oxidized low-density lipoprotein (ox-LDL) has been identified as a key risk factor in the pathogenesis of atherosclerosis[4]. When subjected to irritative stimuli, such as hypertension or dyslipidamia, endothelial and smooth muscle cells express adhesion molecules that promote the local accumulation of ox-LDL[5, 6]. Ox-LDL that has accumulated on the surface of the inner vascular wall may stimulate the expression of many types of cytokines and trigger the development of atherosclerosis[7].

MiRNAs are a family of highly conserved, small non-coding RNAs[8]. These RNAs negatively regulate gene expression by binding to the 3’ untranslated region (3’UTR) of target mRNAs, leading to a reduction in protein expression via degradation or translational inhibition. Emerging evidence has recently shown that several miRNAs play critical roles in the progression of atherosclerosis by influencing the proliferation, migration, and apoptosis of various cell types that line the vascular wall[3, 8]. Recently, several studies have shown that miR-497 is deregulated in many types of tumors and has many biological functions, such as promoting the development of cancer or inducing the multidrug resistance of cancer[9–11]. To reveal the relationship between deregulated miRNAs and early-stage atherosclerosis, we used ApoE-deficient mice to build an animal model of atherosclerosis and analyzed the miRNA expression profiles of the atherosclerotic vascular wall using miRNA microarray analysis. The results showed that miR-497 is significantly upregulated in the arterial wall of the animal model[12]. However, detailed information concerning the role of miR-497 in the development of atherosclerosis is unknown. Recent study has shown that miR-497 in HUVECs induce apoptosis and inhibit the proliferation via targeting VEGFR2/Raf/MEK/ERK signal pathway[13]. But that doesn’t represent it is the only way to affect the functions of ECs.

This study was designed to detect the expression of miR-497 in ox-LDL-treated HUVECs and to investigate the mechanism through which miR-497 affects HUVECs. Our aim was to determine the potential role of miR-497 in vascular endothelial cells during the early stage of atherosclerosis.

## Materials and Methods

### Cell culture and exposure to ox-LDL

HUVECs were purchased from the Shanghai Institute for Biological Sciences, Chinese Academy of Sciences, and cultured in DMEM (Gibco, USA) supplemented with 10% fetal bovine serum (FBS, Gibco, USA), 100 U/ml penicillin, and 100 mg/ml streptomycin. The cells were incubated at 37°C in a humidified chamber supplemented with 5% CO_2_. 3~5 passages of HUVECs were used for further study. HUVECs with the concentration of 10^5^ per well were exposed to 100 μg/ml ox-LDL (Guangzhou Yiyuan Biotech. Co. Ltd) for 0, 3, 6, 12, 24, 36, or 48 h in 6-well plates. The medium was not changed during culture.

### Quantitative real-time PCR (qRT-PCR) analysis of miRNA and mRNA

Total RNA, including small RNAs and mRNA, was extracted using the TRIzol reagent (Invitrogen, USA) according to the manufacturer’s instruction. The concentration of RNA was determined using a NanoDrop spectrophotometer (NanoDrop, USA). The miR-497 stem-loop primer, U6 primer, mRNA primer, and GAPDH primer were bought from Guangzhou RiboBio Co., LTD. The expression of miR-497 was assayed using stem-loop RT, followed by real-time PCR analysis. MiR-497 cDNA was synthesized from total RNA using the miRNA reverse transcription kit (TaKaRa, Dalian, China), and the expression levels of miR-497 were quantified using the miRNA-specific assay kit (TaKaRa, Dalian, China). U6 snRNA was used as an internal control. Reverse transcription of the mRNAs were performed using the PrimeScript RT reagent kit (TaKaRa, Dalian, China), and the expression levels of the mRNAs were determined using SYBR Premix Ex Taq (TaKaRa, Dalian, China), with GAPDH as an internal control. Real-time PCR was performed on a real-time PCR instrument (CFX96, BIORAD, USA). The results of the qRT-PCR analysis were determined based on the threshold cycle (Ct), and the relative expression levels were calculated using the 2^-ΔΔCt^ method, after normalization to the expression of the internal control gene.

### MiR-497 mimics and inhibitor transfection

The miR-497 mimic, mimic-negative, inhibitor, and inhibitor-negative were purchased from Guangzhou RiboBio Co., LTD. For the gain of function analyses, HUVECs were seeded into each well of a 6-well plate and cultured in DMEM containing 10% FBS; then, 50 nM miR-497 mimic or 50 nM miRNA-mimic negative was transfected into the HUVECs using Lipofectamine RNAiMAX (Invitrogen, USA) according to the manufacturer’s protocol. For the loss of function analyses, HUVECs were seeded into each well of a 6-well plate and cultured in DMEM containing 1% FBS without ox-LDL exposion. Then, 100 nM miR-497 inhibitor or 100 nM miRNA-inhibitor negative was transfected into the HUVECs using Lipofectamine RNAiMAX. After 48 hours, the transfected cells were harvested for subsequent experiments. QRT-PCR analysis was used to analyze transfection efficacy.

### Cell proliferation assay

The proliferation of the HUVECs was determined using a CCK-8 assay (Dojindo Molecular Technologies, Inc.) and a 5-ethynyl-2’-deoxyuridine (EdU) assay using an EdU assay kit (Ribobio, Guangzhou, China), as previously described[14, 15]. HUVECs were cultured in 96-well plates at a density of 5×10^3^ cells per well and transfected with the miR-497 mimic, miR-497 inhibitor, or their respective control RNA for 48 h. For the CCK-8 assay, 10 μL of the CCK-8 reagent was added to each well for 2 hours. Then, the absorbance at 450 nm was measured using an Enzyme mark instrument. For the EdU assay, 50 μM EdU was added to the cells, and the cells were incubated for 2 h at 37°C. The cells were then fixed with 4% paraformaldehyde for 15 min at room temperature and exposed to 0.5% Triton X-100 for 20 min. After 3 washes with PBS, the cells were stained with 100 μL of Apollo Dye Solution for 30 min. The nucleic acids in all of the cells were stained with DAPI. Images were taken using a fluorescence microscope (Axio Observer Z1, Carl Zeiss. Inc). All experiments were performed in triplicate.

### Cell cycle assay

After 48 hours of transfection, the cells were harvested, centrifuged at 1200 rpm for 5 min, and washed three times with cold phosphate-buffered saline (PBS). Subsequently, the cells were fixed in 70% ice cold methanol overnight at 4°C. After 30 min digestion with 1 μg/μL RNase, the cells were resuspended in 250 μL of propidium iodide staining solution (10 μg/mL) and incubated for 1 hour at room temperature in the dark. The distribution of G0/G1, S, and G2/M phase cells was determined following analysis on a FACSscan flow cytometer (EPICS XL-MCL, Beckman).

### Apoptosis assay

At 48 hours post-transfection, the cells were harvested and washed three times with PBS, and the total volume of resuspended cells was 5×10^5^; Then, 5 μL of Annexin V-FITC and 5 μL of PI solution were added, and the cells were stained for 15 min in the dark using the Annexin V-FITC Apoptosis Detection kit (KeyGEN Biotech, Nanjing, China). The relative percentage of Annexin V-FITC-positive/PI-negative cells was analyzed by flow cytometry (EPICS XL-MCL, Beckman).

### Caspase-3 and caspase-9 activity assay

The activities of caspase-3 and caspase-9 were measured using a caspase-3 and capsase-9 detection assay kit (Beyotime Institute of Biotechnology, Haimen, Jiangsu, China) according to the manufacturer’s instructions. After treatment, HUVECs were harvested and treated for 15 min with the ice-cold lysis buffer supplied with the kit. The suspensions were then centrifuged at 20000 g for 15 min, and the supernatants were collected. For the detection of caspase-3, 10 μl of supernatant and 10 μl of Ac-DEVD-pNA were added to 80 μl of reaction buffer. For the detection of caspase-9, 10 μl of supernatant and 10 μl of Ac-LEHD-pNA were added to 80 μl of reaction buffer. The mixed samples were incubated at 37°C for 2 hours, and the enzyme-catalyzed released of pNA was quantified at 405 nm using an Enzyme mark instrument.

### Plasmid construction and dual luciferase activity assay

The human Bcl-2 3’-UTR and CCND-2 3’-UTR, which were generated by PCR amplification from HUVEC cell DNA, were cloned into the psiCHECK-2 vector (Promega, Biotech Co., Ltd). The primers for the Bcl-2 3’-UTR were 5'-CCGCTCGAGGATCAGACCTTTGAATGATTC-3' (forward) and 5'-ATAAGAATGCGGCCGCCTCTGTGAATCCCGTTTGAA-3' (reverse). The primers for the CCND-2 3’-UTR were 5'-CCGCTCGAGTCATAGTATGAGGGTTGAAGAC-3' (forward) and 5'- ATAAGAATGCGGCCGCCACACCCATCATCACACAAG-3' (reverse). The miR-497-mut, mimic miR-497, and negative control (NC) were purchased from RiboBio (RiboBio Co. Ltd, Guangzhou, China). HUVECs were seeded in triplicate in 24-well plates and allowed to settle for 24 h. Then, 100 ng of psiCHECK-2-Bcl-2-luciferase, psiCHECK-2-CCND-2-luciferase plasmid, miR497-mut, mimic miR497, or the negative control (NC) was transfected into HUVECs using the Lipofectamine 2000 reagent, according to the manufacturer’s instructions. Luciferase and control signals were measured 48 h after transfection using the Dual Luciferase Reporter Assay Kit (Promega), according to a protocol provided by the manufacturer. All experiments were performed in triplicate.

### Western blot analysis

Western blot analysis was performed as previously described[16]. Briefly, after previous treatment, HUVECs were harvested and homogenized in lysis buffer. Total protein was separated by SDS-PAGE and transferred to PVDF membranes (Millipore Corporation, USA), which were blocked with 5% non-fat milk in Tris-buffered saline and 0.1% Tween 20 for 2 h, followed by incubation with primary antibodies overnight at 4°C. The membranes were then incubated with horseradish peroxidase (HRP)-conjugated secondary antibody for 1 h at room temperature. Finally, the protein bands of interest were visualized using an enhanced chemiluminescence (ECL) detection reagent (General Electric Healthcare company, USA) and were detected using a fluorescence imaging analysis system (ImageQuant^TM^ LAS 4000 mini, General Electric Healthcare company, USA). The primary antibodies for BCL-2, BAX, CCND-2, caspase-3, and α-Tubulin were all purchased from Cell Signaling Technology. A HRP-conjugated anti-rabbit secondary antibody was purchased from General Electric Healthcare Company. The protein levels were normalized to α-tubulin.

### Statistical analysis

The data are expressed as the mean±SD from at least three independent experiments. Student’s t-test and the one-way ANOVA test were employed for statistical analysis. P<0.05 was considered statistically significant. All data were processed using Originpro 8 (OriginLab Corporation, USA) and PASW Statistics 18 (IBM SPSS software, USA).

## Results

### MiR-497 was upregulated in HUVECs treated by ox-LDL

**Fig 1:**
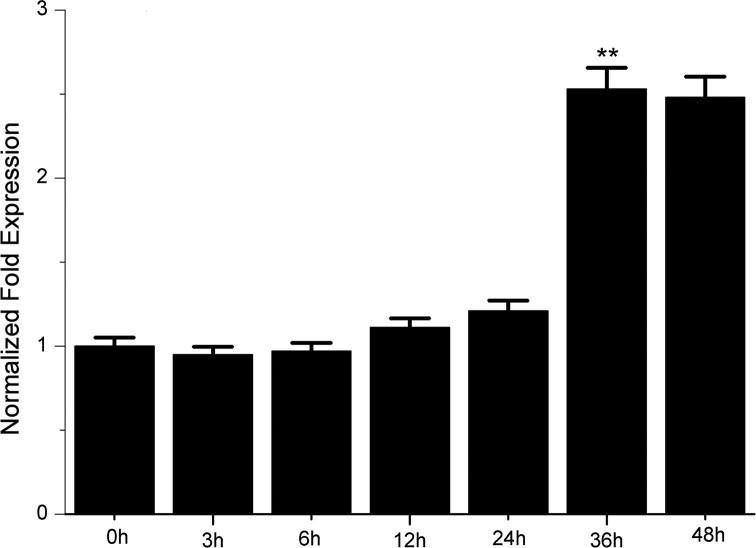
QRT-PCR analysis was used to analyze the levels of miR-497 in HUVECs stimulated with ox-LDL. HUVECs were exposed to 100 μg/ml ox-LDL for 3, 6, 12, 24, 36, or 48 h. The miR-497 levels were significantly upregulated after 36 h of stimulation with 100 μg/ml ox-LDL; **P<0.01.

The expression of miR-497 was examined in HUVECs treated with 100 μg/ml ox-LDL for different time points using qRT-PCR. The results showed that miR-497 was significantly upregulated in the HUVECs after 36 h of treatment with ox-LDL (Fig 1).

### Overexpression of miR-497 inhibited cell proliferation and induced apoptosis

**Fig 2:**
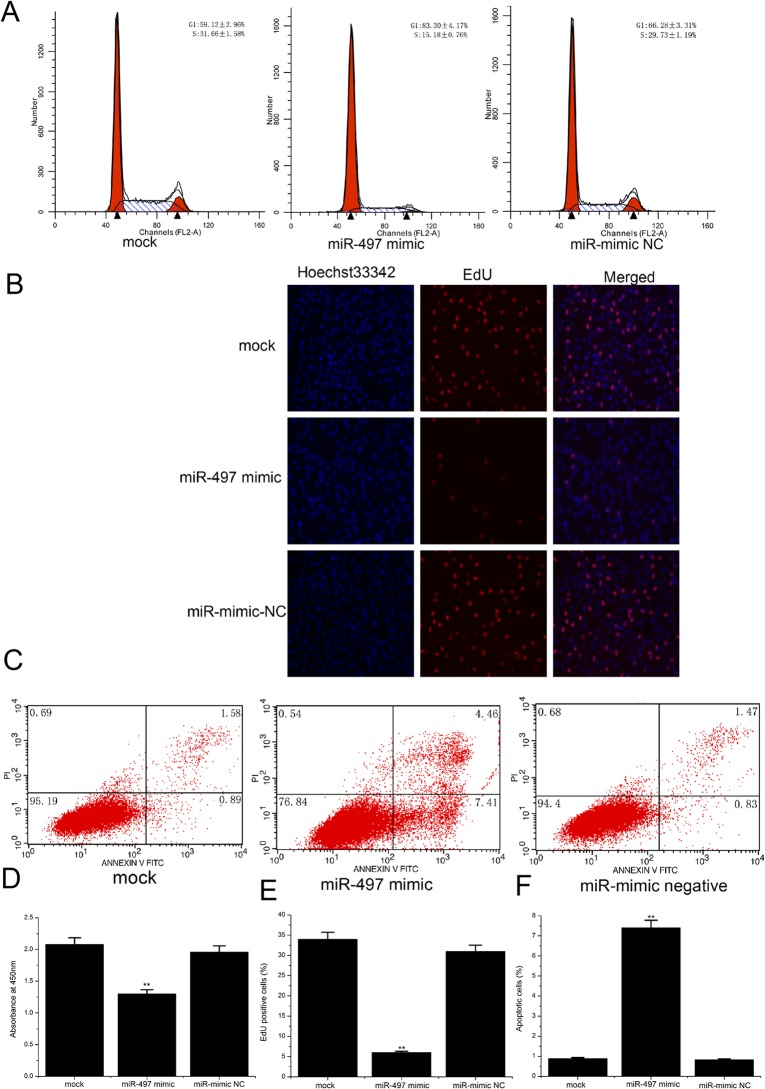
The effects of the overexpression of miR-497 on the proliferation and apoptosis of HUVECs. (A) One representative cell cycle profile of HUVECs detected using a flow cytometer. The cells were stained with PI prior to detection. The overexpression of miR-497 reduced the percentage of cells in the S phase. (B) Representative profiles from the EdU cell proliferation assay after transfection with miR-497 for 48 h (magnification 200×). (E) Amount of EdU-positive cells in the different treatment groups. Increased miR-497 expression inhibited cellular DNA replication in HUVECs. (C) Apoptotic status after transfection, as detected by FCM after Annexin V-FITC/PI labeling. (F) Comparison of apoptotic cells in various groups. The percentage of early apoptotic cells was significantly higher in the experimental group. (D) CCK-8 cell proliferation assay for HUVECs. Cell proliferation was significantly inhibited after miR-497 transfection. The data are presented as the mean ± SD. All results are representative of three independent experiments. **P<0.01 versus the control groups.

To explore the potential role of miR-497 in apoptosis and proliferation of HUVECs, miR-497 mimics or miRNA mimic negative controls (NCs) were used to treat the HUVECs at a concentration of 50 nmol/L for 48 hours. QRT-PCR result showed high transfection efficacy of miR-mimics (S1 Fig). Cell cycle analysis showed that compared with the control group, the experimental group transfected with the miR-497 mimics displayed a significantly higher percentage of cells in the G1 phase (83.3±4.17%) and a lower percentage of cells in the S phase (15.18±0.76%) (Fig 2A). Next, we used the EdU DNA cell proliferation assay, which is a more sensitive and specific approach [17], to evaluate the effects of miR-497 on cell proliferation. The results showed that the percentage of EdU-positive cells was significantly reduced in the experimental group compared with the control group (Fig 2B and 2E). Cell proliferation was also determined using a CCK-8 assay and is expressed as absorbance. Cells transfected with miR-497 apparently grew slower than cells in the control groups (Fig 2D). A flow cytometry assay of apoptosis showed that the expression of miR-497 induced early apoptosis in HUVECs, and the percentage of early apoptotic cells in the experimental group was obviously increased (Fig 2C and 2F). Together, these results demonstrated that overexpression of miR-497 suppressed proliferation and induced the apoptosis of HUVECs.

### Suppression of miR-497 can partially reduce the apoptosis and proliferation inhibition of HUVECs induced by ox-LDL

**Fig 3:**
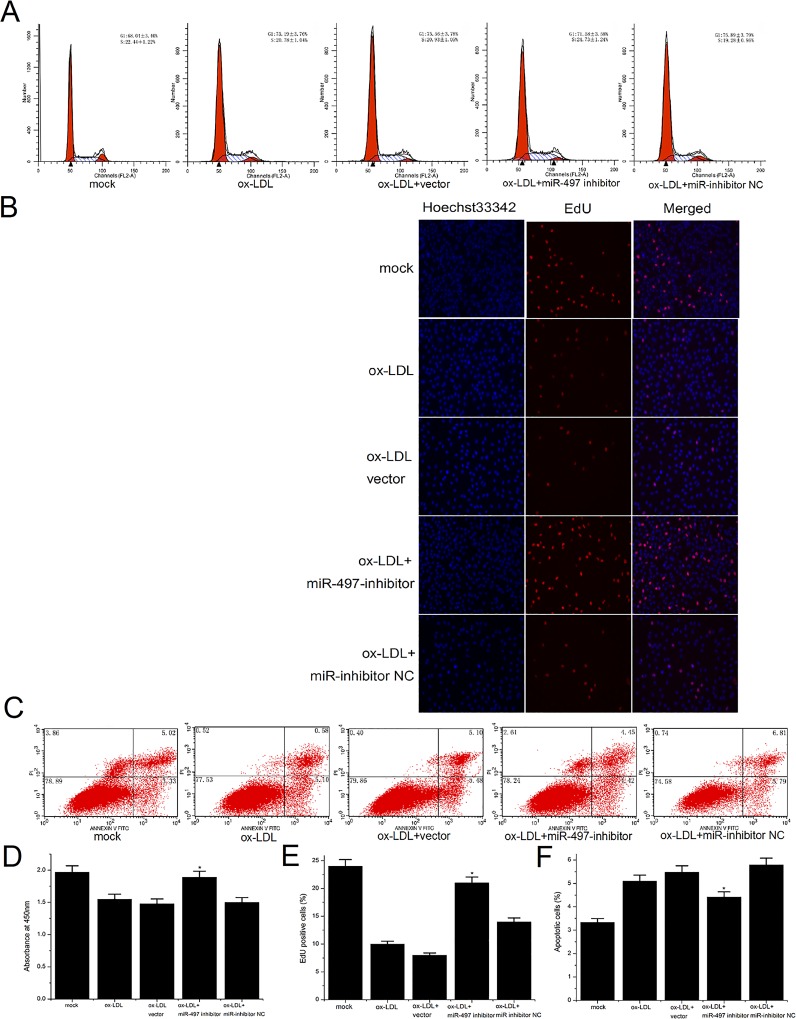
The effects of miR-497 suppression on the proliferation and apoptosis of ox-LDL-induced HUVECs. (A) Cell cycle changes detected by a flow cytometer. The cells were stained with PI prior to detection. Suppression of miR-497 could partially increase the percentage of S-phase cells that were reduced by the addition of ox-LDL. (B) EdU cell proliferation analysis after transfection, as detected using a fluorescence microscope (magnification 200×). (C) Apoptotic status after transfection, as detected by FCM after Annexin V-FITC/PI labeling. (D) CCK-8 cell proliferation assay for HUVECs. Suppression of miR-497 could partially reduce the inhibition of cell proliferation induced by ox-LDL. (E) Rate of EdU-positive cells in the different groups. Loss of miR-497 can partially reverse the reduction of cellular DNA replication in HUVECs stimulated with ox-LDL. (F) Comparison of apoptotic cells in the various groups. Inhibition of miR-497 could partially reduce the number of apoptotic cells induced by ox-LDL. The data are presented as the mean ± SD. All results shown are representative of three independent experiments. *P<0.05 and **P<0.01 versus the control groups.

To explore the functional roles of suppressed miR-497 in ox-LDL-induced HUVECs, the cells were exposed to 100 μg/ml ox-LDL and were transfected with 100 nmol/L miR-497 inhibitors or miRNA negative NCs for 48 hours. QRT-PCR result showed high transfection efficacy of miR-inhibitors (S2 Fig). The inhibition of proliferation and the induction of apoptosis were detected in the HUVECs following ox-LDL stimulation. A cell cycle analysis showed that the miR-497 inhibitor could partially reduce the percentage of G1-phase cells and increase the percentage of S-phase cells in the treatment group induced by ox-LDL (Fig 3A). The EdU DNA cell proliferation assay showed that the percentage of EdU-positive cells in the experimental group treated with ox-LDL and the miR-497 inhibitor was significantly increased compared with the control group treated with ox-LDL only or in combination with the miR-inhibitor NC (Fig 3B and 3E). The CCK-8 assay was also used to test cell proliferation. The results showed that loss of miR-497 could partially reduce the proliferation inhibition induced by ox-LDL (Fig 3D). Early apoptosis was detected by annexin-V/propidium iodide staining, followed by flow cytometry analysis. The results showed that the miR-497 inhibitor significantly decreased the percentage of apoptotic cells following ox-LDL treatment (Fig 3C and 3F). Together, these results demonstrated that the miR-497 inhibitor can protect HUVECs from ox-LDL-induced proliferation inhibition and apoptosis.

### Identification of Bcl-2 and CCND-2 as potential target genes of miR-497

**Fig 4:**
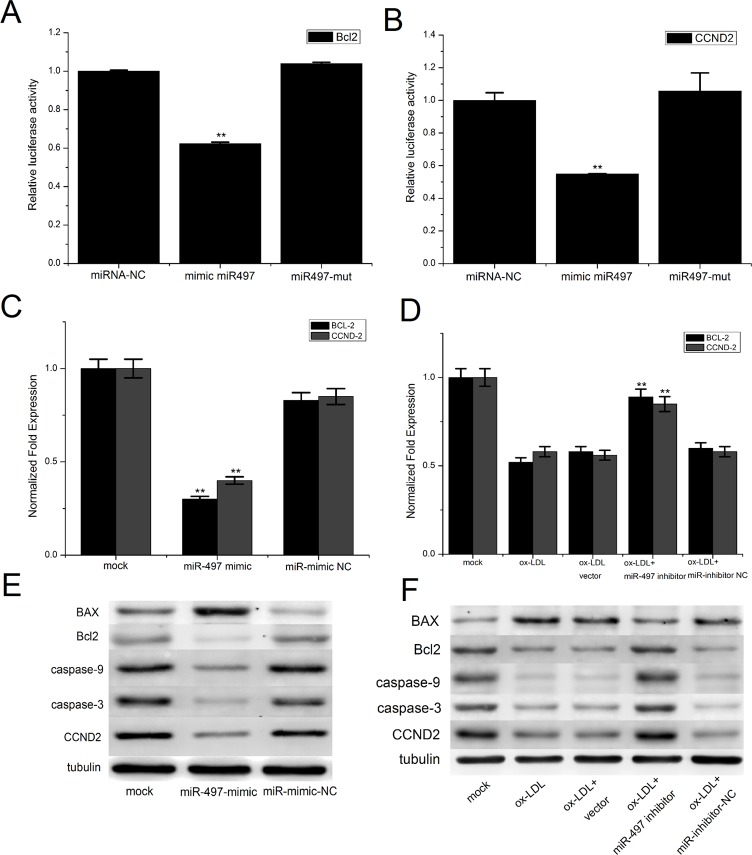
Bcl2 and CCND2 are target genes of miR-497 in HUVECs. (A) Luciferase assay in HUVECs co-transfected with miR-497 or miR-497-mutant and a luciferase reporter containing the Bcl2 3’UTR. (B) Luciferase assay in HUVECs co-transfected with miR-497 or miR-497-mutant and a luciferase reporter containing the CCND2 3’UTR. (C) Bcl2 mRNA was analyzed using qRT-PCR after transfection with miR-497 or the miR-497 inhibitor. (D) CCND2 mRNA was analyzed using qRT-PCR after transfection with miR-497 or the miR-497 inhibitor. (E) The Bcl2/Bax, CCND2, and caspase-3 and -9 proteins were analyzed using western blot analysis after transfection with miR-497. (F) The Bcl2/Bax, CCND2, and caspase-3 and -9 proteins were analyzed using western blot analysis after transfection with miR-497 inhibitor. The data are presented as the mean ± SD from three separate experiments. **P<0.01 versus the control groups.

The target genes were first predicted using the TargetScan and miRanda programs. After the bioinformatics analyses (S3 Fig), we identified BCL2 and CCND2 as possible direct target genes that are involved in the proliferation and apoptosis of HUVECs. To verify the prediction, we cloned the 3’UTRs of the Bcl2 and CCND2 genes into a luciferase reporter vector, and these constructs were co-transfected into HUVECs with plasmids expressing wild-type or mutant miR-497. The results showed that wild-type miR-497 significantly inhibited the luciferase activity of the Bcl2 and CCND2 3’UTR, but the mutant miR-497 did not (Fig 4A and 4B). Furthermore, the overexpression of miR-497 could significantly downregulate the levels of Bcl2 and CCND2 mRNA and protein in HUVECs (Fig 4C and 4E). Suppression of miR-497 could partially reduce the downregulation in the Bcl2 and CCND2 mRNA and protein levels in HUVECs induced with ox-LDL (Fig 4D and 4F). These results indicated that Bcl2 and CCND2 are direct target genes of miR-497.

### Overexpression of miR-497 increased caspase-9/3 activity

**Fig 5:**
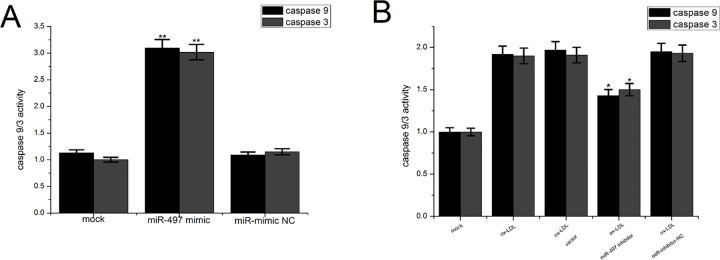
The effects of miR-497 on caspase-9/3 activities. (A) The caspase-9/3 activities in the HUVECs were increased after transfection of miR-497. (B) Inhibition of miR-497 could partially reduce the caspase-9/3 activities that were increased following ox-LDL treatment in HUVECs. The data are presented as the mean ± SD from three separate experiments.*P<0.05 and **P<0.01 versus the control groups.

To examine the mechanism of apoptosis that is induced by miR-497, we transfected miR-497 into HUVECs and examined the activities of caspase-9 and -3. The results showed that overexpression of miR-497 significantly increased the activities of caspase-9/3 in the HUVECs (Fig 5A). Western blot analysis showed that ectopic expression of miR-497 could reduce the levels of caspase-9/3, which indicates that caspase-9 and -3 were activated after transfection with miR-497 (Fig 4E).

### Suppression of miR-497 can partially decrease the caspase-9/3 activity in HUVECs induced by ox-LDL

To further examine the role of miR-497 in the apoptosis of HUVECs, we used HUVECs treated by ox-LDL to simulate the state of endothelial cells during the development of atherosclerosis; these cells were then transfected with the miR-497 inhibitor.A caspase-9/3 activity assay showed that the miR-497 inhibitor could partially suppress the caspase-9/3 activation induced by ox-LDL (Fig 5B). Western blot analysis showed that the suppression of miR-497 expression could partially reduce the cleavage of the caspase-9/3 proteins (Fig 4F). Together, these results demonstrated that miR-497 induced apoptosis in the HUVECs through the Bcl2/Bax-caspase-9-caspase-3 pathway.

## Discussion

Endothelial dysfunction is one of the initial steps in the development of atherosclerosis[18, 19]. When exposed to various insults, such as oxidative stress, ECs become activated and express adhesion molecules, gradually resulting in certain pathological changes, including apoptosis and proliferation inhibition[5, 20]. Ox-LDL has been shown to be an important risk factor in the initiation and progression of atherosclerosis[21]. It can induce the apoptosis of ECs and promote the formation of foam cells[22]. Therefore, we used ox-LDL to stimulate HUVECs to simulate the pathological changes that occur in ECs during the early stage of atherosclerosis.

MiRNAs, which typically bind directly to the 3’UTR of their target mRNAs, are important small, non-coding RNAs that participate in the regulation of target gene expression. One miRNA may target multiple genes, and one gene also might be targeted by multiple miRNAs. Recently, miRNAs have been found to be important regulators of EC function during the development of atherosclerosis^[^^3^^]^. Proliferation, apoptosis, senescence, and migration can be regulated by miR-101, miR-126, miR-217, and miR-320^[^^23^^–^^26^^]^. Vascular inflammation might be regulated by miR-19a and miR-125[27, 28]. Thus, miRNAs and their target genes might provide potential therapeutic strategies for the treatment of atherosclerosis. Previously, we utilized an miRNA microarray to analyze the miRNA expression profile of arterial walls with early-stage atherosclerosis in a mouse model[12]. The results showed that miR-497 was one of the most highly upregulated miRNAs, but its detailed role in the development of atherosclerosis was unknown.

In this study, we examined the expression levels of miR-497 in HUVECs stimulated with ox-LDL. The results of the qRT-PCR analysis showed that miR-497 was significantly upregulated in the ox-LDL-induced HUVECs compared with the control group (Fig 1). This result suggests that miR-497 might participate in the endothelial dysfunction in atherosclerosis. Other studies have reported that deregulated miR-497 is associated with apoptosis, proliferation, and the drug resistance of tumor cells[9, 11]. Apoptosis and proliferation of ECs are important pathogenic events in the formation of atherosclerosis[5, 21]. Therefore, we hypothesized that miR-497 might be involved in the development of atherosclerosis by regulating the apoptosis and proliferation of ECs. Further studies have shown that overexpression of miR-497 significantly induced apoptosis and inhibited the proliferation of HUVECs, and suppression of miR-497 could partially reduce the apoptosis and proliferation inhibition induced by ox-LDL (Figs 2 and 3). Therefore, we confirmed that miR-497 played an important role in the regulation of ox-LDL-induced apoptosis and proliferation inhibition. These results demonstrated that miR-497 might participate in atherosclerosis, in part by affecting the apoptosis and proliferation of ECs.

The caspase and Bcl-2 protein families play crucial roles in the development of apoptosis. Caspase-3 has been considered to be the major executive caspase in apoptosis[29]. It operates as the key enzyme in the mitochondria-dependent apoptosis pathway[30]. Therefore, measurement of caspase-3 activity is the major and the most reliable determining factor for apoptosis[31]. The Bcl-2 protein families included both pro-apoptotic (e.g., Bax) and anti-apoptotic (e.g., Bcl-2) members[32]. The anti-apoptotic protein Bcl-2 can regulate apoptosis through caspase-9 and -3-dependent pathways[33–35]. Increased Bax and decreased Bcl-2 expression can stimulate the release of cytochrome c from the mitochondria, which activates caspase-9[36]. Caspase-9 then catalyzes the activation of caspase-3, ultimately leading to apoptosis[37, 38]. CCND2 is an important cell cycle gene that induces G0/G1 arrest, leading to the inhibition of cell proliferation[39]. Deregulated CCND2 has been reported in various diseases and has been implicated in cell proliferation[40, 41]. In our study, the dual luciferase activity assay demonstrated that miR-497 could directly bind to both the Bcl2 and CCND2 3’UTRs. qRT-PCR and western blot analyses showed that miR-497 negatively regulated both Bcl2 and CCND2 expression at the mRNA and protein levels. The levels and activities of the caspase-9/3 proteins were increased after the overexpression of miR-497 and were decreased when the overexpression of miR-497 was suppressed in HUVECs induced with ox-LDL. Based on these results, we confirmed that miR-497 regulated the apoptosis of HUVECs through the Bcl-2-caspase-9-caspase3 pathway. Ectopic expression of miR-497 regulated cell cycle progression and proliferation in HUVECs through the action of CCND2.

In conclusion, the present study showed that miR-497 is upregulated in HUVECs stimulated with ox-LDL. Increased miR-497 expression can induce apoptosis and suppress proliferation, whereas the loss of miR-497 expression can partially reduce the apoptosis and proliferation inhibition induced by ox-LDL. The results of the dual luciferase reporter assay and the inverse correlation between miR-497 and Bcl2 or CCND2 expression indicated that Bcl2 and CCND2 are direct target genes of miR-497 in HUVECs. Further study showed that miR-497 induced the apoptosis of HUVECs through the Bcl2/Bax-caspase-9-caspase-3 pathway. The present results indicated that miR-497 might take part in vascular endothelial cell injury during the early stages of atherosclerosis and could be a potential therapeutic target for the treatment of atherosclerosis.

## Supporting Information

S1 Fig Transfection efficacy of miR-mimics analyzed by qRT-PCR. (TIF)

S2 Fig Transfection efficacy of miR-inhibitors analyzed by qRT-PCR. (TIF)

S3 Fig Bioinformatics analyses of target genes by TargetScan and miRanda programs. (TIF)
