# Supplementary figures and images for: MicroRNA-497 Induces Apoptosis and Suppresses Proliferation via the Bcl-2/Bax-Caspase9-Caspase3 Pathway and Cyclin D2 Protein in HUVECs
Source: PLoS One. 2016 Dec 5;11(12):e0167052. doi: 10.1371/journal.pone.0167052 (PMC5137897; doi:10.1371/journal.pone.0167052)

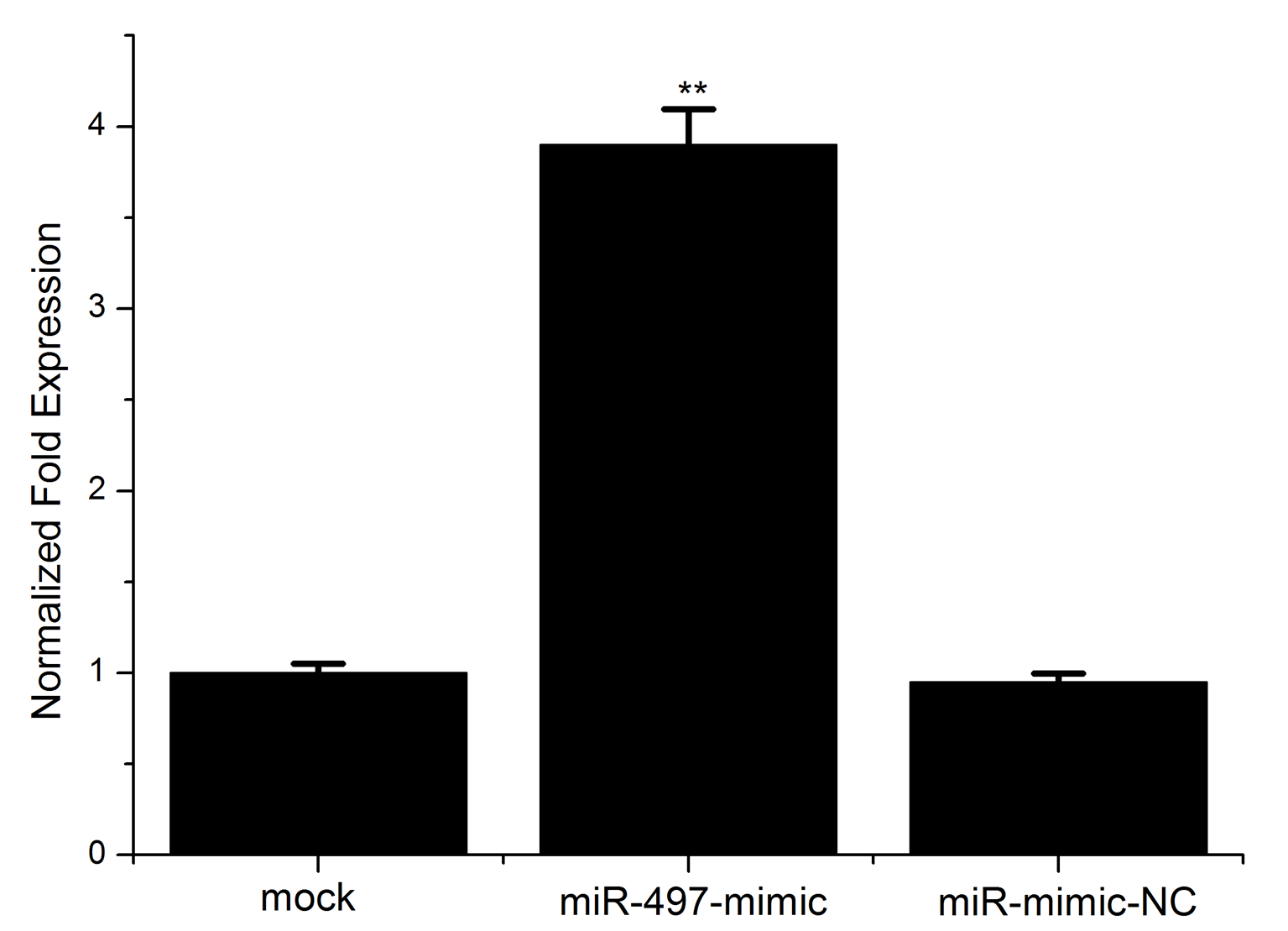

Supplement: S1 Fig — (TIF) [file pone.0167052.s001.tif]

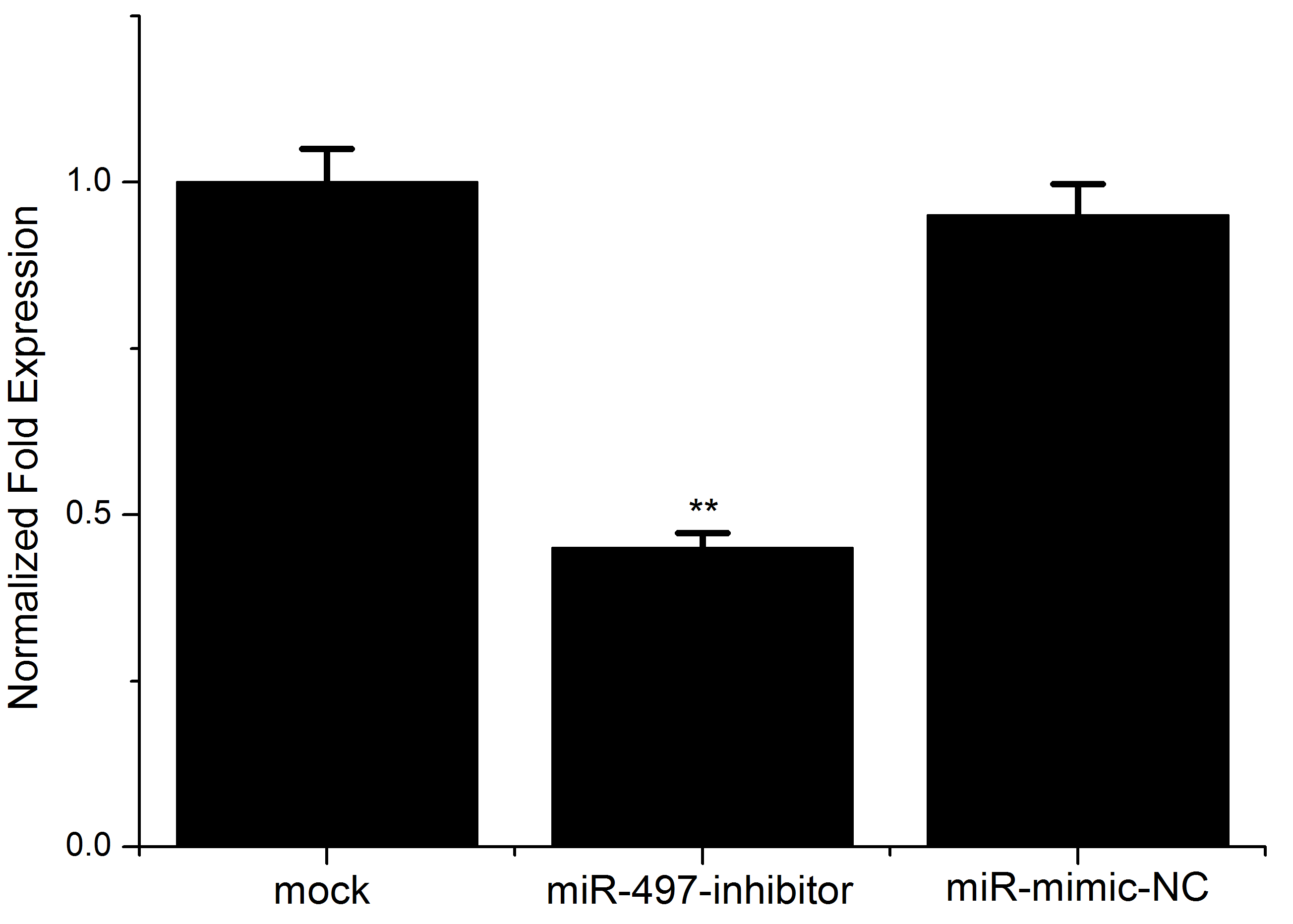

Supplement: S2 Fig — (TIF) [file pone.0167052.s002.tif]

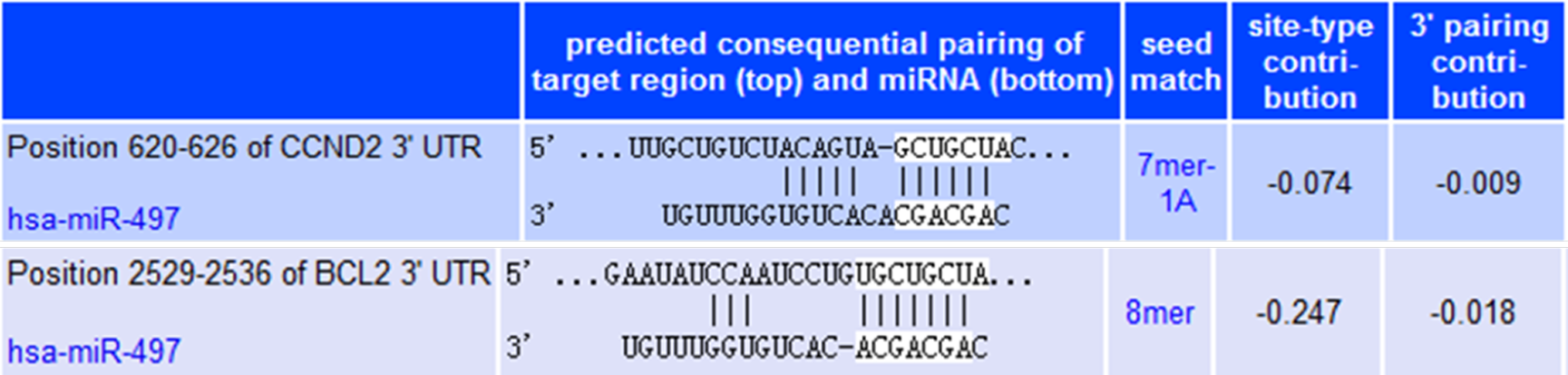

Supplement: S3 Fig — (TIF) [file pone.0167052.s003.tif]
